# Editorial: Angiogenesis and access to vasculature as a target in gastrointestinal tumors and predictive biomarkers identification: an open challenge

**DOI:** 10.3389/fonc.2024.1428174

**Published:** 2024-05-20

**Authors:** Eleonora Lai, Pina Ziranu, Francesco Pezzella, Andrea Pretta, Nicole Liscia, Zhiwei Hu

**Affiliations:** ^1^ Medical Oncology Unit, University Hospital and University of Cagliari, Cagliari, Italy; ^2^ Nuffield Division of Clinical Laboratory Science-Radcliffe Department of Medicine (NDCLS-RDM) John Radcliffe Hospital, University of Oxford, Oxford, United Kingdom; ^3^ Department of Medical Oncology, Istituto di Ricovero e Cura a Carattere Scientifico (IRCCS) San Raffaele Scientific Institute, Vita-Salute San Raffaele University, Milan, Italy; ^4^ Department of Surgery, Division of Surgical Oncology, The Ohio State University Wexner Medical Center and James Comprehensive Cancer Center, Columbus, OH, United States

**Keywords:** tumor angiogenesis, gastrointestinal cancer, predictive biomarkers, anti-angiogenic agents, treatment resistance

The Research Topic “Angiogenesis and Access to Vasculature as a Target in Gastrointestinal Tumors and Predictive Biomarkers Identification: an Open Challenge” was launched to explore the complex landscape of tumor-driven angiogenesis and non-angiogenic growth in gastrointestinal cancers, as well as potential biomarkers for anti-angiogenic agents, and to provide new evidence on the identification of clinical and translational prognostic and predictive factors in this setting. The aim of the research was to improve patients’ selection for anti-angiogenic treatments and thus their clinical outcomes.

Early detection and treatment of cancers including gastrointestinal cancers is essential in halting tumor progression and saving the lives of cancer patients. Tumor angiogenesis, formation of pathological neovasculature in tumor microenvironment, is an early event in tumorigenesis (1) and one of the hallmarks of cancer (2), whereby tumor cells acquire nutrients and grow rapidly and form metastases. Under hypoxic tumor microenvironment and consequence of genetic mutations, transcription factor hypoxia-inducible factors (HIF) can be induced in cancer stem cells/cancer cells followed by secretion of potent angiogenic growth factors, such as vascular endothelial growth factor (VEGF), and other angiogenesis-related molecules and cells, eventually leading to formation of tumor neovasculature and expression of new surface angiogenic receptors (for example, tissue factor) (3–5), which can be potentially used for development of new anti-neovasculature and/or angiogenesis blockage therapies (6). Indeed, angiogenesis and/or co-option of normal vessels plays a crucial role in gastrointestinal cancers development, growth, metastatic spread, and survival. For this reason, tumor-driven angiogenesis represents one of the most important therapeutic targets for the treatment of advanced gastrointestinal tumors (7). Currently, a consistent number of anti-angiogenic agents have been developed and are now available for these patients across different lines of treatment (7, 8). Unfortunately, not all patients respond to anti-angiogenic drugs and some of them develop resistance, thus experiencing unnecessary potential toxicities. To date, despite extensive research, no validated clinical/translational predictive factors to identify patients who are more likely to positively respond to anti-angiogenic treatment are available. Therefore, identifying predictive factors is essential for optimizing treatment potentialities, avoiding unnecessary toxicities, and improving patients’ survival. A huge research effort has been made over the years in order to find a reliable predictive factor among tissue-based genetic polymorphisms, circulating biomarkers, circulating tumor cells and ctDNA, miRNAs, and more recently on imaging tools (including but not limited to radiomics) (9).

Our goal was to provide a deeper understanding of the mechanisms of resistance to anti-angiogenic drugs in gastrointestinal cancer and resources to overcome treatment resistance, by focusing on the state of the art of anti-angiogenic treatment in gastrointestinal tumors, on new evidence, and on future clinical and translational perspectives.

Our Research Topic included one original article, two mini-reviews and one review.

Jiang at al. assessed the roles of insulin-like growth factor-II mRNA-binding protein 3 (IGF2BP3) in hypoxia-induced cell migration and angiogenesis and its N6-methyladenosine (m6A)-dependent targets in gastric cancer *in vitro* and *in vivo*. Indeed, IGF2BP3 includes HIF1A among its targets and can positively regulate its expression in gastric cancer cells through a direct binding to a specific m6A site in HIF1A mRNA. The Authors demonstrated that IGF2BP3 knockdown was able to inhibit hypoxia-induced cell migration and angiogenesis by down-regulating HIF1A in gastric tumors.

In their minireview, Sultana et al. provided a comprehensive overview on human and mouse angiogenins, focusing on their structure and its correlation with their function, as well as their involvement in tumor angiogenesis but also in other cancer-related processes. Indeed, angiogenin is a well-known angiogenic factor and its role in gastrointestinal tumors has been demonstrated. Thus, a deeper knowledge of human and mouse angiogenin might provide more information on potential therapeutic targets and pharmaceutical development in the near future.

Ebeling et al. analyzed in their review the pathways involved in the regulation of tumor angiogenesis by the crosstalk between innate immunity and endothelial cells during tumor progression. Moreover, they discussed the potential contribution of the interaction among myeloid cells and innate lymphocytes with endothelial cells in the tumor microenvironment for the development of anti-cancer treatments. More specifically, they analyzed the crosstalk between tumor-associated macrophages and endothelial cells, the angiogenic features of circulating myeloid cells, the role of NK cells in cancer angiogenesis, helper-like innate lymphoid cells and endothelial cells activation mediated by TNF, IL-8 and TGF-b.

Lei et al. reported the clinical cases of two colorectal cancer patients and one gastric cancer patient with MSS/pMMR status who were treated in later lines with a combination of apatinib, camrelizumab and trifluridine/tipiracil followed by apatinib maintenance. Then, they reviewed the literature evidence on the association of anti-angiogenic treatment plus immunotherapy to explore the potential role of acute inflammatory reaction during anti-angiogenic therapy plus immunotherapy as a possible indicator of therapeutic effect. As editors of the inaugural Frontiers “Angiogenesis and Access to Vasculature as a Target in Gastrointestinal Tumors and Predictive Biomarkers Identification: an Open Challenge” article collection, we would like to thank our contributing authors for their research effort and for providing new insights in gastrointestinal cancer angiogenesis. We thank the contributing Authors also for providing starting points for further investigation on this challenging topic, with the aim to eventually identify new therapeutic targets and/or more reliable predictive factor for anti-angiogenic treatment response to improve patients’ outcome.

## Author contributions

EL: Writing – original draft, Writing – review & editing. PZ: Writing – original draft, Writing – review & editing. FP: Writing – original draft, Writing – review & editing. AP: Writing – original draft, Writing – review & editing. NL: Writing – original draft, Writing – review & editing. ZH: Writing – original draft, Writing – review & editing.
